# Deciphering cancer heterogeneity: the biological space

**DOI:** 10.3389/fcell.2014.00012

**Published:** 2014-04-03

**Authors:** Stephanie Roessler, Anuradha Budhu, Xin W. Wang

**Affiliations:** Laboratory of Human Carcinogenesis, Center for Cancer Research, National Cancer InstituteBethesda, MD, USA

**Keywords:** integrated genomics, primary liver cancer, hepatocellular carcinoma, cancer genomic heterogeneity, cancer drivers, gene signatures

## Abstract

Most lethal solid tumors including hepatocellular carcinoma (HCC) are considered incurable due to extensive heterogeneity in clinical presentation and tumor biology. Tumor heterogeneity may result from different cells of origin, patient ethnicity, etiology, underlying disease, and diversity of genomic and epigenomic changes which drive tumor development. Cancer genomic heterogeneity thereby impedes treatment options and poses a significant challenge to cancer management. Studies of the HCC genome have revealed that although various genomic signatures identified in different HCC subgroups share a common prognosis, each carries unique molecular changes which are linked to different sets of cancer hallmarks whose misregulation has been proposed by Hanahan and Weinberg to be essential for tumorigenesis. We hypothesize that these specific sets of cancer hallmarks collectively occupy different tumor biological space representing the misregulation of different biological processes. In principle, a combination of different cancer hallmarks can result in new convergent molecular networks that are unique to each tumor subgroup and represent ideal druggable targets. Due to the ability of the tumor to adapt to external factors such as treatment or changes in the tumor microenvironment, the tumor biological space is elastic. Our ability to identify distinct groups of cancer patients with similar tumor biology who are most likely to respond to a specific therapy would have a significant impact on improving patient outcome. It is currently a challenge to identify a particular hallmark or a newly emerged convergent molecular network for a particular tumor. Thus, it is anticipated that the integration of multiple levels of data such as genomic mutations, somatic copy number aberration, gene expression, proteomics, and metabolomics, may help us grasp the tumor biological space occupied by each individual, leading to improved therapeutic intervention and outcome.

Like other lethal solid tumors, most primary liver cancer patients are considered incurable due to extensive heterogeneity in clinical presentation and tumor biology. Thereby, tumor heterogeneity may result from different cells of origin, the range in patient ethnicity, etiology, underlying disease and diversity of genomic and epigenomic changes, which drive tumor development (Shen and Laird, 2013). Molecular differences between tumors from different patients, inter-tumor heterogeneity, and between different areas of an individual tumor, intra-tumor heterogeneity, have been recognized, possibly emanating from the presence of cancer stem cells or selection by clonal evolution (Nguyen et al., 2012). Cancer genomic heterogeneity thereby results in varying degrees of clinical presentation and tumor biology, which impedes treatment options and poses a significant challenge to cancer management. Despite great progress in the development of new treatment modalities, the improvement of cancer mortality is very modest, especially for common lethal cancers such as esophageal, liver, lung, and pancreatic cancer (American Cancer Society, 2012). Molecularly targeted therapies are promising new treatment options, however, they may not fundamentally reduce overall mortality in an unstratified cohort. For example, the first-line treatment for advanced hepatocellular carcinoma (HCC), sorafenib, a small molecular inhibitor of several tyrosine protein kinases, provides only a 2.8 month improvement of overall median survival (Llovet et al., 2008). On the other hand, selection of patients that may respond to a specific treatment may lead to greatly improved outcome. For example, the tyrosine kinase inhibitor gefitinib has been shown to be effective in a select group of non-small cell lung cancer patients with epidermal growth factor receptor (EGFR) mutations, providing a 13.5 month improvement of median overall survival (Takano et al., 2005). Interferon-alpha is effective in preventing tumor relapse in a select group of HCC patients with reduced expression of miR-26 in tumor cells, with an estimated improvement of median overall survival of more than 7-years (Ji et al., 2009). Therefore, the major hurdle in fundamentally improving cancer patient outcome is tumor heterogeneity. This is evident by the fact that current treatment modalities appear to only be effective in a small group of patients with select biological alterations underlying the considerable influence of inter and intra-tumor heterogeneity on clinical advances. Thus, our ability to identify distinct groups of cancer patients with similar tumor biology who are most likely to respond to a specific therapy would have a significant impact on improving patient outcome.

To address this problem, molecular-based technologies including genomic, transcriptomic, proteomic, and metabolomic profiling, have been applied to clinical specimens of multiple cancer types with the aim of identifying distinct tumor subgroups with unique tumor biology, which house critical and specific alterations in gatekeepers of cancer initiation and progression. Consequently, various genome-based signatures have been developed as diagnostic or prognostic tools to discriminate patients with inter-tumor heterogeneity, assist in molecular re-staging or predict outcome. These studies, which include matched case control specimens, homogenous patient populations of the same etiology and independent cohorts for validation, have aided in the successful identification of specific genomic aberrations necessary for tumor growth and maintenance. Examples include signatures linked to metastasis, tumor recurrence, inactivation of specific tumor suppressor genes such as TP53 and cancer stem cells (Lee et al., 2004; Budhu et al., 2006; Yamashita et al., 2009; Woo et al., 2011). In addition, molecularly-guided technologies might complement diagnostic classification, allowing physicians to predict patient outcome and to select the most appropriate treatment for each cancer patient. These approaches would allow us to triage patients into homogeneous groups, each of which is linked to specific genomic aberrations with biological implications, which may respond to a particular treatment (Roessler et al., 2007; Kumar et al., 2011).

Over the last 10 years, multiple prognostic gene signatures within the same cancer type have emerged from omics studies, which raise questions about their biological significance. Are certain gene signatures identifying particular patient subgroups with the same or different molecular traits? We examined this problem in the context of HCC as we and others have developed multiple prognostic gene signatures within the same cohort of HCC patients, which have been validated in independent patient cohorts (Table 1 and Supplemental Information). To this end, we compared 7 different gene signatures that are individually associated with survival in the same HCC cohort. The first gene signature we assessed consists of 153 genes associated with portal venous metastases (Ye et al., 2003). This signature not only predicts metastasis at the time of diagnosis, but also recurrence within 2 years after diagnosis in early stage patients (Roessler et al., 2010). A second gene signature, a driver gene signature, was recently developed by integrating transcriptomic and somatic copy number aberration profiling and consists of 10 genes that show correlation between gene expression and chromosomal alteration (Roessler et al., 2012). The third signature is a 65-gene based risk score classifier built by the overlap of a proliferation and a recurrence signature (Kim et al., 2012). A fourth signature composed of 770 genes was developed by comparison of patients associated with miR-26 expression (Ji et al., 2009), while the fifth signature was related to fatty acid metabolism (FAM; *n* = 273) (Budhu et al., 2013). Two additional unpublished gene signatures were included, an unsupervised survival signature not restricted to any phenotype (Cox proportional hazards model; *n* = 336; *p* < 0.001) and a hepatic stem cell-like signature (*n* = 932; Zhao et al., unpublished). A comparison of these 7 signatures shows that they are mainly associated with different altered pathways and cellular functions. From this result, we would expect that the signatures predict different patient subgroups linked to perturbations of their corresponding pathways. To test this hypothesis, we performed subgroup prediction using the 7 gene signatures followed by hierarchical clustering of the prediction results. We found that patients largely separated into two clusters with concordant survival prediction (Figure 1A). The driver signature tends to assign high risk of poor outcome to more patients when compared to the other signatures. Thus, a group of patients has high risk only according to this signature. It appears that the gene signatures are superior to TP53 mutation, tumor size and clinical staging. As expected, the genes of the above 7 gene signatures did not show significant overlap (hypergeometric test; alpha < 0.05; Figure 1B). Thus, despite variances in gene alteration among these signatures, they seem to differentiate concordant patient outcome groups.

**Table 1:** **Seven outcome-related HCC gene signatures**.

**Classifier**	**Main function**	**Genes**	**Key genes**	**References**
FAM	Fatty acid metabolism	273	SCD	Budhu et al., 2006, 2013
Metastasis	Early recurrence	160	OPN	Ye et al., 2003; Roessler et al., 2012
Driver genes	Survival	10	8p genes, DLC1 etc.	Roessler et al., 2012
Proliferation	Proliferation	65	CTNNB1, AKT	Kim et al., 2012
miR-26	Inflammation	770	NF-kB/IL-6	Ji et al., 2009
Stem-like	Stem cell function	932	EpCAM	Zhao et al. (unpublished)
Survival	Survival	336		Current study

**Figure 1:**
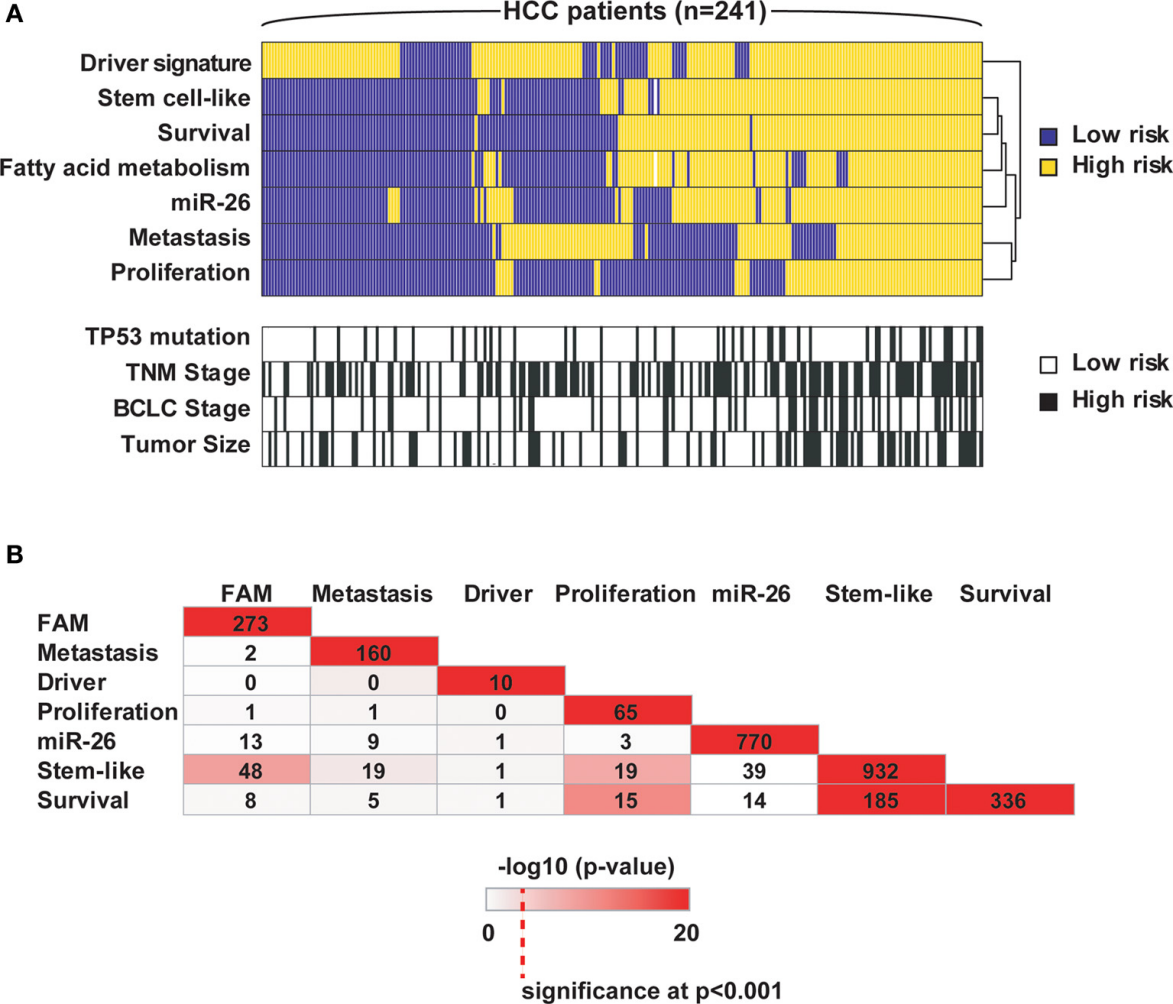
**Seven independent gene signatures predict concordant outcome groups. (A)** Each of the 241 HCC patients (columns) was assigned into high and low risk groups based on hierarchical clustering of the prediction results of the 7 independent gene signatures, TP53 mutation status (wild type vs. mutated), tumor size (<5 cm vs. >5 cm), TNM staging (I vs. II/III) and BCLC staging (A vs. B/C); each tumor subclassification (rows) based on the clustering results was found to be independently associated with prognosis based on Cox Regression model. High risk, poor survival; low risk, better survival. **(B)** The numbers represent the overlap of genes among the 7 signatures. Color intensities represent the negative log10 hypergeometric *p*-values. A color intensity scale bar and the significance level at *p* < 0.001 are indicated.

The high heterogeneity observed in the HCC population would have suggested that multiple patient subgroups exist, each of which share similar tumor biology. Ten cancer hallmarks have been suggested to be required for tumorigenesis: sustaining proliferative signaling, evading growth suppressors, resisting cell death, enabling replicative immortality, inducing angiogenesis, activating invasion, avoiding immune destruction, tumor-promoting inflammation, deregulating cellular energetics and genome instability and mutation (Hanahan and Weinberg, 2011). Thereby, each tumor exhibits a subset of cancer hallmarks at differing degrees, which hypothetically might be ideal therapeutic targets. However, if each gene signature predicts the same poor outcome group, what is the main cancer hallmark that is affected and what is the ideal treatment for this patient subgroup? Joan Massagué introduced the concept of prognostic space, which refers to the range of prognostic possibilities specified by a particular prognostic indicator (Massague, 2007). Thereby, distinct gene signatures that do not show any overlap at the gene level may reflect a common set of phenotypic traits where each trait is defined by a set of gene-expression events. Thus, although signatures may be largely different from one another, they may occupy overlapping prognostic space, indicative of similar outcome (Massague, 2007). Due to the observed tumor heterogeneity within the same prognostic subgroup shared by various gene signatures, it is unlikely that all patients in a poor outcome group will benefit from the same treatment. Moreover, patient heterogeneity may not simply be reflected by a certain gene signature, since similar gene expression patterns might also be induced by different molecular mechanisms. For example, not all patients with predicted stem cell features may benefit from the same treatment because the stem cell features might have arisen due to perturbation of different molecular pathways. This suggests that convergent evolution of tumors may lead to the development of similar gene expression patterns by independent molecular mechanisms, perhaps due to adaptation to similar environmental conditions. Thus, the multidimensional composition of cancer hallmarks in each tumor needs to be pinpointed so that a rationally-designed treatment regimen can be introduced.

We suggest an expansion of the prognostic space model into what we term a biological space model, which incorporates tumor heterogeneity (Figure 2). In this modified model, patient subgroups are represented as planets, which reside on orbits corresponding to their key signaling pathways. Although patient subgroups may share a common prognosis, the driving signaling pathway, whether singular or multiple, may have a significant effect on the resultant composition of the tumor, and hence its placement in the biological space. Moreover, the dynamic nature of this model, represented by the size, shape and width of the signaling pathway orbits, takes into account the important role of tumor plasticity, which allows tumors to adapt to selective pressure due to treatment or tumor environmental changes such as increased nutrient demand in the growing tumor mass. Thus, over time the position of a patient subgroup (planet) may change representing for example the development of resistance to a certain drug. This revised model builds upon the premise that although signatures share a common prognostic space, each carries unique molecular changes, which are linked to different sets of cancer hallmarks that collectively occupy different tumor biological space.

**Figure 2:**
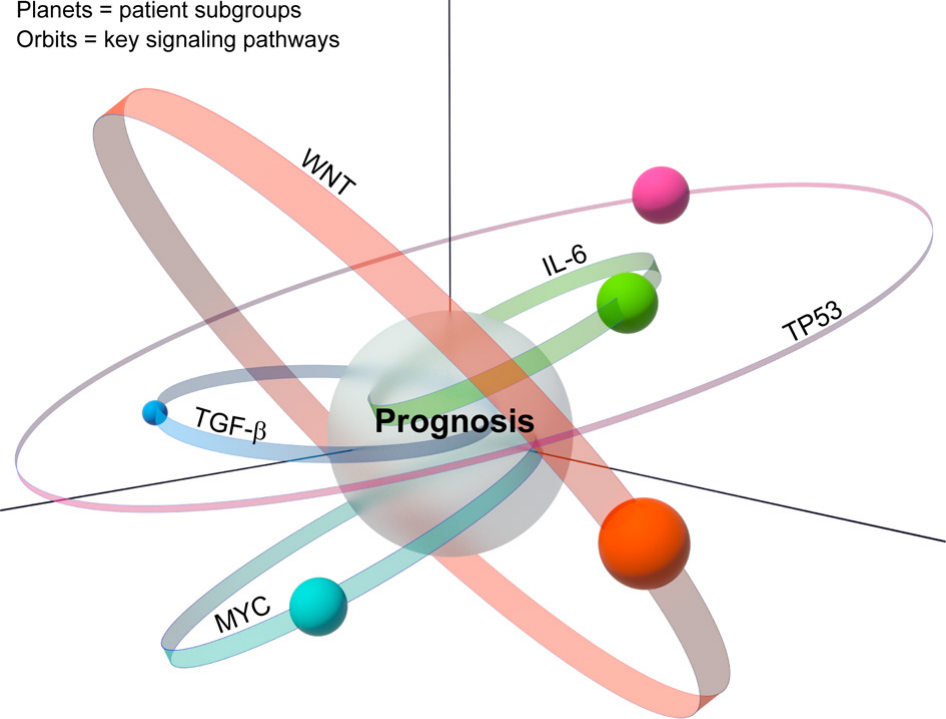
**A model depicts the relationship between the biological space and the prognostic space.** Multiple patient subgroups exist because of tumor heterogeneity. Analogous to planets, various patients' subgroups reside on orbits, each of which carries key unique signaling pathways. Representative altered signaling pathways such as MYC, TP53, etc, are indicated. The patients' subgroups whose orbits intersect with the prognostic space are considered those with similar prognostic outcome.

Our progress in understanding the mechanisms of cancer development and progression over the last three decades, has led to the development of mechanism-based therapies to treat cancer patients. Ideally, targeted therapeutics will be developed for each of the 10 cancer hallmarks. However, it is currently a challenge to identify a particular hallmark or a newly emerged molecular network as a therapeutic target for a particular tumor. Recent efforts of the Pan-Cancer initiative on the first 12 tumor types collected and analyzed by The Cancer Genome Atlas (TCGA) have begun to lay a framework to assemble coherent, consistent TCGA data sets across tumor types and across platforms (The Cancer Genome Atlas Research Network et al., 2013). Consequently, the list of cancer-associated genes has grown rapidly with the large sample size attained by TCGA. However, the search across different cancer types shows that increased sample size may also increase the number of false positive genes due to heterogeneity in mutation frequency (Lawrence et al., 2013, 2014; The Cancer Genome Atlas Research Network et al., 2013). A recent study in breast cancer analyzing driver mutations and copy number changes in cancer driver genes showed that the number of mutated driver genes varies greatly between individual patients (Stephens et al., 2012). Of the 40 cancer driver genes, 33 genes, each of which is altered relatively infrequently, were responsible for 42% of the driving genetic events. Thus, many infrequently mutated genes collectively and substantially contribute to tumor development, making it very challenging to select the best treatment option for each cancer patient. In addition, it is challenging to identify specific hallmarks because one particular genetic change does not always reflect cancer vulnerability. For instance, a gene mutation may not imply that tumor survival depends on this gene, although the mutation may be important in the early genesis of this tumor. Furthermore, different combinations of affected genes may give rise to a different dependency for a particular tumor subgroup. One strategy to search for cancer vulnerabilities is to use *in vivo* screens such as transposon mutagenesis or RNAi (Zender et al., 2008; Bard-Chapeau et al., 2013). The limitation is that the findings are based on animal models, which may or may not be relevant to human tumor subtypes. Conceivably, integrative analysis incorporating biological and clinical factors will be necessary to tease apart differences in tumors, rooted in heterogeneity, to identify critical biomarkers for cancer diagnosis and clinically relevant therapeutic targets that represent convergent cancer driving molecular hubs. Therefore, the TCGA Pan-Cancer initiative seeks to incorporate multiple omics data with genomic sequencing (The Cancer Genome Atlas Research Network et al., 2013). Such an approach requires the development of new bioinformatics tools. For example, we have developed an approach to integrate somatic copy number alterations and transcriptome to identify novel tumor suppressor genes in HCC (Roessler et al., 2012). We also integrated mRNA and microRNA expression profiles and identified key signaling pathway in stem-cell like intrahepatic cholangiocarcinoma (Oishi et al., 2012). Integration of metabolomic and mRNA expression profiles resulted in the identification of a key lipogenic pathway and the druggable target SCD in HCC (Budhu et al., 2013). Most recently, we developed an unsupervised tumor subgroup discovery tool, namely iSubgraph, which is based on graph mining and mixture models, to integrate various omics-based data (Ozdemir et al., 2013). It is anticipated that the integration of multiple levels of data such as genomic mutations, somatic copy number aberration, gene expression, proteomics, and metabolomics, may help us grasp the tumor biological space occupied by each individual. The dynamic nature of the biological space of each tumor takes into account the important role of tumor plasticity which is the main reason for the development of drug resistance. Therefore, it will be crucial not only to assess the main cancer driving event, but also to predict which resistance mechanisms a tumor might develop in order to select suitable drug combinations, which can lead to improved therapeutic intervention and outcome. We believe that *in vivo* mutagenesis screens to identify cancer drivers in animal tumor models would complement efforts from integrative omics based profiling of patient biospecimens to facilitate the identification of molecular tumor subgroups and cancer vulnerable molecular targets.

## Author contributions

Stephanie Roessler, Anuradha Budhu and Xin W. Wang initiated the concept, designed the study, analyzed and interpreted the data, and wrote the manuscript.

## Conflict of interest statement

The authors declare that the research was conducted in the absence of any commercial or financial relationships that could be construed as a potential conflict of interest.
