# Supplementary material for: Deciphering cancer heterogeneity: the biological space
Source: Front Cell Dev Biol. 2014 Apr 3;2:12. doi: 10.3389/fcell.2014.00012 (PMC4207029; doi:10.3389/fcell.2014.00012)
Supplement: Supplementary file 1 [file Presentation1.PDF]

Supplemental Information

Deciphering cancer heterogeneity: the biological space

Stephanie Roessler, Anuradha Budhu, Xin Wei Wang

Supplemental Table 1: Metastasis gene signature. Of the 153 genes identified originally [1], 146 genes are present on the HG-U133A Affymetrix microarray platform.

Rank	Gene symbol	Entrez Gene ID	Map Location	Defined Genelist
1	IVNS1ABP	10625	1q25.1-q31.1	
2	SPP1	6696	4q21-q25	Regulators of Bone Mineralization, Cell Communication, ECM-receptor interaction, Focal adhesion, immunology
3	IRF2	3660	4q34.1-q35.1	gene_regulation, transcription
4	RPS3	6188	11q13.3-q13.5	Ribosome
5	CES1	1066	16q13-q22.1	Alkaloid biosynthesis II, immunology
6	IL2RB	3560	22q13|22q13.1	IL 2 signaling pathway, IL-2 Receptor Beta Chain in T cell Activation, Cytokine-cytokine receptor interaction, Jak-STAT signaling pathway, immunology
7	SELENBP1	8991	1q21-q22	
8	CALCOCO2	10241	17q21.32	
9	RAD50	10111	5q31	ATM Signaling Pathway, Role of BRCA1, BRCA2 and ATR in Cancer Susceptibility, DNA_replication, misc
10	FEN1	2237	11q12	DNA_replication
11	LANCL2	55915	7q31.1-q31.33	
12	RGS20	8601	8q	
13	RAB8A	4218	19p13.1	Rab GTPases Mark Targets In The Endocytotic Machinery
14	GCLC	2729	6p12	Glutamate metabolism, Glutathione metabolism
15	STUB1	10273	16p13.3	
16	CHEK1	1111	11q24-q24	ATM Signaling Pathway, cdc25 and chk1 Regulatory Pathway in response to DNA damage, Cell Cycle: G2/M Checkpoint, RB Tumor Suppressor/Checkpoint Signaling in response to DNA damage, Regulation of cell cycle progression by Plk3, Role of BRCA1, BRCA2 and ATR in Cancer Susceptibility, Cell cycle, cell_cycle, cell_signaling, signal_transduction
17	CD37	951	19q13.3	Hematopoietic cell lineage, angiogenesis, metastasis
18	CDH13	1012	16q24.2-q24.3	cell_signaling, metastasis
19	ZNF415	55786	19q13.41	

20	CDCA4	55038	14q32.33	
21	DNAJC9	23234	10q22.2	
22	CENPE	1062	4q24-q25	gene_regulation, transcription
23	ENO2	2026	12p13	Glycolysis / Gluconeogenesis, Phenylalanine, tyrosine and tryptophan biosynthesis
24	KHK	3795	2p23.3	Fructose and mannose metabolism, immunology
25	U2AF1	7307	21q22.3	Spliceosomal Assembly, immunology
26	GTF2H4	2968	6p21.3	Basal transcription factors
27	UGT8	7368	4q26	Sphingolipid metabolism, pharmacology
28	JOSD1	9929	22q13.1	
29	RFTN1	23180	3p24.3	
30	DTYMK	1841	2q37.3	Pyrimidine metabolism
31	AKR1C4	1109	10p15-p14	Androgen and estrogen metabolism, Bile acid biosynthesis, C21-Steroid hormone metabolism, Metabolism of xenobiotics by cytochrome P450
32	TRMU	55687	22q13	
33	PDLIM1	9124	10q22-q26.3	
34	CD247	919	1q22-q23	Activation of Csk by cAMP-dependent Protein Kinase Inhibits Signaling through the T Cell Receptor, CTL mediated immune response against target cells , HIV Induced T Cell Apoptosis, IL 17 Signaling Pathway, IL12 and Stat4 Dependent Signaling Pathway in Th1 Development, Lck and Fyn tyrosine kinases in initiation of TCR Activation, NO2-dependent IL 12 Pathway in NK cells, Role of Tob in T-cell activation, Stathmin and breast cancer resistance to antimicrotubule agents, T Cell Receptor and CD3 Complex, T Cell Receptor Signaling Pathway, T Cytotoxic Cell Surface Molecules, T Helper Cell Surface Molecules, The Co-Stimulatory Signal During T-cell Activation, Natural killer cell mediated cytotoxicity, T cell receptor signaling pathway
35	RFC5	5985	12q24.2-q24.3	DNA polymerase, Purine metabolism, Pyrimidine metabolism
36	CSF3R	1441	1p35-p34.3	Cytokine-cytokine receptor interaction, Hematopoietic cell lineage, Jak-STAT signaling pathway, immunology
37	PDCD11	22984	10q24.33	
38	CENTD3	64411	5q31.3	
39	SLC12A8	84561	3q21.2	
40	UNC119B	84747	12q24.31	
41	RPS6KA4	8986	11q11-q13	MAPKinase Signaling Pathway, MAPK signaling pathway
42	ARPP-21			
43	USP12	219333	13q12.13	
44	GALK1	2584	17q24	Leloir pathway of galactose metabolism, Galactose metabolism, immunology
45	C2orf3	6936	2p11.2-p11.1	
46	SPTBN2	6712	11q13	
47	RAB3A	5864	19p13.2	Rab GTPases Mark Targets In The Endocytotic Machinery, tsonc
48	SPG11			
49	SERPINB6	5269	6p25	

50	LTF	4057	3p21.31	Perou's- Intrinsic- Breast-Cancer-Genes, immunology
51	SUV420H1	51111	11q13.2	
52	MYD88	4615	3p22	Inactivation of Gsk3 by AKT causes accumulation of b-catenin in Alveolar Macrophages, NFkB activation by Nontypeable Hemophilus influenzae, NF-kB Signaling Pathway, Signal transduction through IL1R, Toll-Like Receptor Pathway, Apoptosis, Toll-like receptor signaling pathway, apoptosis, immunology, misc
53	FZD2	2535	17q21.1	Colorectal cancer, Wnt signaling pathway, development
54	NOL7	51406	6p23	
55	GPD2	2820	2q24.1	Electron Transport Reaction in Mitochondria, Glycerophospholipid metabolism, immunology
56	NR1D2	9975	3p24.2	
57	FUT8	2530	14q24.3	Glycan structures - biosynthesis 1, Keratan sulfate biosynthesis, N-Glycan biosynthesis
58	HSD3B1	3283	1p13.1	Androgen and estrogen metabolism, C21-Steroid hormone metabolism, immunology
59	SART1	9092	11q13.1	
60	ASPH	444	8q12.1	Hypoxia-Inducible Factor in the Cardiovascular System, immunology
61	HOXB13	10481	17q21.2	
62	GCNT2	2651	6p24.2	Glycan structures - biosynthesis 2, Glycosphingolipid biosynthesis - neo-lactoseries
63	COL4A5	1287	Xq22	
64	EMD	2010	Xq28	
65	ATP6V1G2	534	6p21.3	ATP synthesis, Epithelial cell signaling in Helicobacter pylori infection, Oxidative phosphorylation
66	LPHN1	22859	19p13.2	
67	GUCA2B	2981	1p34-p33	
68	CCR3	1232	3p21.3	CCR3 signaling in Eosinophils, IL 5 Signaling Pathway, Selective expression of chemokine receptors during T-cell polarization, The Role of Eosinophils in the Chemokine Network of Allergy, Cytokine-cytokine receptor interaction, immunology
69	BASP1	10409	5p15.1-p14	
70	ADD2	119	2p14-p13	
71	KIAA0319L	79932	1p34.2	Perou's- Intrinsic- Breast-Cancer-Genes
72	EPB42	2038	15q15-q21	Anthrax Toxin Mechanism of Action, immunology, misc
73	CDS1	1040	4q21.23	Glycerophospholipid metabolism, Phosphatidylinositol signaling system
74	ANKRD1	27063	10q23.31	
75	PITX2	5308	4q25-q27	Multi-step Regulation of Transcription by Pitx2, TGF-beta signaling pathway, development, misc, transcription
76	SPOCK3	50859	4q32.3	
77	EFTUD1	79631	15q25.2	
78	LOC151162	151162	2q21.3	
79	PYGB	5834	20p11.2-p11.1	Insulin signaling pathway, Starch and sucrose metabolism
80	ABL2	27	1q24-q25	NA, cell_cycle, cell_signaling, signal_transduction, tsonc
81	TBC1D16	125058	17q25.3	

82	SLC20A2	6575	8p12-p11	immunology
83	MATK	4145	19p13.3	Epithelial cell signaling in Helicobacter pylori infection, NA, Regulation of actin cytoskeleton, cell_cycle, cell_signaling, signal_transduction
84	AP1S1	1174	7q22.1	
85	RHAG	6005	6p21.1-p11	
86	N4BP1	9683	16q12.1	
87	ATF2	1386	2q32	ALK in cardiac myocytes, Angiotensin II mediated activation of JNK Pathway via Pyk2 dependent signaling, MAPKinase Signaling Pathway, p38 MAPK Signaling Pathway , The 4-1BB-dependent immune response, The information-processing pathway at the IFN-beta enhancer, MAPK signaling pathway
88	STOML1	9399	15q24-q25	
89	MMP9	4318	20q11.2-q13.1	Inhibition of Matrix Metalloproteinases, Leukocyte transendothelial migration, angiogenesis, immunology
90	ZC3H12A	80149	1p34.3	
91	GRHL2	79977	8q22.3	
92	MAP3K6	9064	1p36.11	MAPKinase Signaling Pathway, MAPK signaling pathway
93	PDK1	5163	2q31.1	AKT Signaling Pathway, Inactivation of Gsk3 by AKT causes accumulation of b-catenin in Alveolar Macrophages, mTOR Signaling Pathway, Phosphoinositides and their downstream targets., PTEN dependent cell cycle arrest and apoptosis, Regulation of eIF4e and p70 S6 Kinase, Skeletal muscle hypertrophy is regulated via AKT/mTOR pathway, Fc epsilon RI signaling pathway, T cell receptor signaling pathway
94	CRIP1	1396	14q32.33	
95	SLTM	79811	15q22.1	
96	NUP205	23165	7q33	
97	LIMK1	3984	7q11.23	Axon guidance, Regulation of actin cytoskeleton, cell_cycle, cell_signaling, signal_transduction
98	YES1	7525	18p11.31-p11.21	Adherens junction, NA, Tight junction, signal_transduction, tsonc
99	HIPK1	204851	1p13.2	
100	CTRB1	1504	16q23-q24.1	
101	C14orf102	55051	14q32.11	
102	TRAP1	10131	16p13.3	
103	COPA			ADP-Ribosylation Factor, Neuroactive ligand-receptor interaction
104	NCAPD3	23310	11q25	
105	PRUNE	58497	1q21	Purine metabolism
106	KCNQ1	3784	11p15.5	immunology
107	CLCN7	1186	16p13	
108	ENO3	2027	17pter-p11	Glycolysis / Gluconeogenesis, Phenylalanine, tyrosine and tryptophan biosynthesis
109	HCG2P7	80867	6p21.3	
110	GTF2H1	2965	11p15.1-p14	Basal transcription factors
111	PRR3	80742	6p21.33	

112	G3BP2	9908	4q21.1	
113	RAB28	9364	4p15.33	
114	VPS41	27072	7p14-p13	
115	PLA2G6	8398	22q13.1	Aspirin Blocks Signaling Pathway Involved in Platelet Activation, Eicosanoid Metabolism, Fc Epsilon Receptor I Signaling in Mast Cells, p38 MAPK Signaling Pathway , Arachidonic acid metabolism, Fc epsilon RI signaling pathway, Glycerophospholipid metabolism, GnRH signaling pathway, Linoleic acid metabolism, Long-term depression, MAPK signaling pathway, VEGF signaling pathway
116	PLD2	5338	17p13.1	Metabolism of Anandamide, an Endogenous Cannabinoid, Glycerophospholipid metabolism, GnRH signaling pathway
117	TGOLN2	10618	2p11.2	
118	PMAIP1	5366	18q21.32	
119	ITGA9	3680	3p21.3	Cell adhesion molecules (CAMs), ECM-receptor interaction, Focal adhesion, Regulation of actin cytoskeleton, cell_signaling, metastasis
120	PCF11	51585	11q13	
121	GYS1	2997	19q13.3	Insulin signaling pathway, Starch and sucrose metabolism, immunology
122	HMMR	3161	5q33.2-qter	ECM-receptor interaction, cell_signaling, metastasis
123	LY6E	4061	8q24.3	cell_cycle, cell_signaling
124	ZNF646	9726	16p11.2	
125	YRDC	79693	1p34.3	
126	NFKB1	4790	4q24	Acetylation and Deacetylation of RelA in The Nucleus, Activation of PKC through G protein coupled receptor, AKT Signaling Pathway, ATM Signaling Pathway, Bone Remodelling, Cadmium induces DNA synthesis and proliferation in macrophages, CD40L Signaling Pathway, Ceramide Signaling Pathway, Chaperones modulate interferon Signaling Pathway, Corticosteroids and cardioprotection, CXCR4 Signaling Pathway, Double Stranded RNA Induced Gene Expression, Erythropoietin mediated neuroprotection through NF-kB, fMLP induced chemokine gene expression in HMC-1 cells, Free Radical Induced Apoptosis, HIV-I Nef: negative effector of Fas and TNF, Human Cytomegalovirus and Map Kinase Pathways, Inactivation of Gsk3 by AKT causes accumulation of b-catenin in Alveolar Macrophages, Induction of apoptosis through DR3 and DR4/5 Death Receptors , Influence of Ras and Rho proteins on G1 to S Transition, Keratinocyte Differentiation, MAPKinase Signaling Pathway, Neuropeptides VIP and PACAP inhibit the apoptosis of a ...
127	WWTR1	25937	3q23-q24	
128	CRISPLD2	83716	16q24.1	
129	TRIM33	51592	1p13.1	
130	ALDH3B2	222	11q13	Glycolysis / Gluconeogenesis, Histidine metabolism, Metabolism of xenobiotics by cytochrome P450, Phenylalanine metabolism, Tyrosine metabolism
131	SH3GLB1	51100	1p22	1- and 2-Methylnaphthalene degradation, Alkaloid biosynthesis II, Benzoate degradation via CoA ligation, Ethylbenzene degradation, Glycerophospholipid metabolism, Limonene and

				pinene degradation, Phenylalanine metabolism, Tyrosine metabolism, Valine, leucine and isoleucine degradation
132	LRP6			Wnt/LRP6 Signalling, Wnt signaling pathway
133	SUPT7L	9913	2pter-p25.1	
134	RPA2	6118	1p35	DNA_replication
135	FANCC	2176	9q22.3	BRCA1-dependent Ub-ligase activity, Role of BRCA1, BRCA2 and ATR in Cancer Susceptibility, DNA_damage, immunology
136	LPL	4023	8p22	Low-density lipoprotein (LDL) pathway during atherogenesis, Mechanism of Gene Regulation by Peroxisome Proliferators via PPARa(alpha), Role of PPAR-gamma Coactivators in Obesity and Thermogenesis, Visceral Fat Deposits and the Metabolic Syndrome, Alzheimer\'s disease, Glycerolipid metabolism, PPAR signaling pathway, immunology
137	TACC1	6867	8p11	
138	GABRE	2564	Xq28	Perou's- Intrinsic- Breast-Cancer-Genes, Neuroactive ligand-receptor interaction
139	MTUS1	57509	8p22	
140	LRPPRC	10128	2p21	IL 6 signaling pathway, Role of ERBB2 in Signal Transduction and Oncology
141	MVK	4598	12q24	Biosynthesis of steroids
142	MOAP1	64112	14q32	
143	ALDH3B1	221	11q13	Glycolysis / Gluconeogenesis, Histidine metabolism, Metabolism of xenobiotics by cytochrome P450, Phenylalanine metabolism, Tyrosine metabolism
144	CHGB	1114	20pter-p12	Perou's- Intrinsic- Breast-Cancer-Genes, misc
145	MDFI	4188	6p21	
146	VAMP3	9341	1p36.23	SNARE interactions in vesicular transport

Supplemental Table 2: Cox regression analysis reveals 336 survival associated genes at p<0.001.

Rank	p-value	Gene symbol	Entrez Gene ID	Map Location	DefinedGenelist
1	2.00E-07	IVNS1ABP	10625	1q25.1-q31.1	
2	4.00E-07	SOX4	6659	6p22.3	gene_regulation, immunology, transcription
3	8.00E-07	HAGH	3029	16p13.3	Pyruvate metabolism, immunology
4	9.00E-07	CCDC44	51204	17q23.3	
5	1.70E-06	NEDD9	4739	6p25-p24	
6	2.00E-06	PLOD2	5352	3q23-q24	Perou's- Intrinsic- Breast-Cancer-Genes, Lysine degradation
7	2.50E-06	SERPINE1	5054	7q21.3-q22	Fibrinolysis Pathway, Platelet Amyloid Precursor Protein Pathway, Complement and coagulation cascades
8	2.90E-06	CAT	847	11p13	The IGF-1 Receptor and Longevity, Amyotrophic lateral sclerosis (ALS), Methane metabolism, Tryptophan metabolism, immunology, pharmacology
9	3.00E-06	PSME1	5720	14q11.2	Antigen processing and presentation, cell_signaling
10	4.50E-06	ZNF124			transcription
11	4.80E-06	CYLD	1540	16q12.1	
12	5.20E-06	SLC27A5	10998	19q13.43	PPAR signaling pathway
13	6.60E-06	TTC31	64427	2p13.1	
14	7.10E-06	EHHADH	1962	3q26.3-q28	Mechanism of Gene Regulation by Peroxisome Proliferators via PPARa(alpha), Benzoate degradation via CoA ligation, beta-Alanine metabolism, Butanoate metabolism, Caprolactam degradation, Fatty acid metabolism, Limonene and pinene degradation, Lysine degradation, Propanoate metabolism, Tryptophan metabolism, Valine, leucine and isoleucine degradation
15	7.90E-06	AQP9	366	15q22.1-q22.2	
16	8.20E-06	APOC4	346	19q13.2	
17	9.40E-06	NDUFA4L2	56901	12q13.3	Oxidative phosphorylation
18	9.70E-06	CYP7A1	1581	8q11-q12	FXR and LXR Regulation of Cholesterol Metabolism, Nuclear Receptors in Lipid Metabolism and Toxicity, Bile acid biosynthesis, PPAR signaling pathway, immunology
19	1.00E-05	SLC10A1	6554	14q24.1	
20	1.02E-05	MAST3	23031	19p13.11	
21	1.06E-05	ADAMTS5	11096	21q21.3	

22	1.14E-05	ATOX1	475	5q32	Perou's- Intrinsic- Breast-Cancer-Genes, immunology
23	1.37E-05	SPP2	6694	2q37-qter	
24	1.48E-05	DHRS1	115817	14q12	1- and 2-Methylnaphthalene degradation, Benzoate degradation via CoA ligation, Bisphenol A degradation, Ethylbenzene degradation, gamma-Hexachlorocyclohexane degradation, Limonene and pinene degradation
25	1.51E-05	SUOX	6821	12q13.2	Sulfur metabolism, immunology
26	1.54E-05	SORBS2	8470	4q35.1	
27	1.55E-05	GPLD1	2822	6p22.3-p22.2	ADP-Ribosylation Factor, Glycosylphosphatidylinositol(GPI)-anchor biosynthesis
28	1.55E-05	SULT2A1	6822	19q13.3	Androgen and estrogen metabolism, Sulfur metabolism, immunology
29	1.58E-05	SLC6A4	6532	17q11.1-q12	immunology
30	1.61E-05	NTS	4922	12q21	Neuroactive ligand-receptor interaction
31	1.72E-05	HEY1	23462	8q21	
32	1.78E-05	PID1	55022	2q36.3	
33	1.79E-05	FMO4	2329	1q23-q25	
34	2.19E-05	CYP3A43	64816	7q21.1	gamma-Hexachlorocyclohexane degradation, Linoleic acid metabolism, Metabolism of xenobiotics by cytochrome P450
35	2.21E-05	RAB8B	51762	15q22.2	
36	2.28E-05	DCXR	51181	17q25.3	Pentose and glucuronate interconversions
37	2.43E-05	MARCH2			
38	2.59E-05	IFIT1	3434	10q25-q26	
39	2.73E-05	ARHGEF2	9181	1q21-q22	
40	2.77E-05	SLC4A1	6521	17q21-q22	immunology
41	2.81E-05	CLIC1	1192	6p22.1-p21.2	
42	3.02E-05	RTP4	64108	3q27.3	
43	3.08E-05	CD14	929	5q22-q32|5q31.1	Inactivation of Gsk3 by AKT causes accumulation of b-catenin in Alveolar Macrophages, Toll-Like Receptor Pathway, Hematopoietic cell lineage, MAPK signaling pathway, Regulation of actin cytoskeleton, Toll-like receptor signaling pathway, angiogenesis, immunology, metastasis, misc

44	3.12E-05	HSD17B4	3295	5q21	Perou's- Intrinsic- Breast-Cancer-Genes, Mechanism of Gene Regulation by Peroxisome Proliferators via PPARa(alpha), Butanoate metabolism, Caprolactam degradation, Fatty acid elongation in mitochondria, Fatty acid metabolism, Lysine degradation, PPAR signaling pathway, Tryptophan metabolism, Valine, leucine and isoleucine degradation
45	3.33E-05	APOL3	80833	22q13.1	
46	3.52E-05	SNRPF	6636	12q22	Spliceosomal Assembly
47	3.58E-05	ACSM2B			
48	3.77E-05	GNG7	2788	19p13.3	
49	3.79E-05	TLR6	10333	4p14	Toll-like receptor signaling pathway
50	3.87E-05	ETNK2	55224	1q32.1	
51	4.61E-05	ADH1B	125	4q21-q23	1- and 2-Methylnaphthalene degradation, Bile acid biosynthesis, Fatty acid metabolism, Glycerolipid metabolism, Glycolysis / Gluconeogenesis, Metabolism of xenobiotics by cytochrome P450, Tyrosine metabolism
52	4.63E-05	MASP2	10747	1p36.3-p36.2	Complement Pathway, Lectin Induced Complement Pathway, Complement and coagulation cascades
53	4.67E-05	AQP8	343	16p12	
54	4.76E-05	UPB1	51733	22q11.2	beta-Alanine metabolism, Pantothenate and CoA biosynthesis, Pyrimidine metabolism
55	5.12E-05	CDKN1C	1028	11p15.5	Cell cycle, cell_cycle, immunology, tsonc
56	5.33E-05	ECHDC2	55268	1p32.3	Perou's- Intrinsic- Breast-Cancer-Genes
57	5.81E-05	CNGA1	1259	4p12-cen	immunology
58	6.23E-05	PIPOX	51268	17q11.2	Glycine, serine and threonine metabolism, Lysine degradation
59	6.52E-05	SLC39A1	27173	1q21	
60	6.57E-05	APBB3	10307	5q31	
61	6.74E-05	ATG4A	115201	Xq22.1-q22.3	
62	6.86E-05	FARP2			Adherens junction, Focal adhesion
63	6.93E-05	GOT2	2806	16q21	Alanine and aspartate metabolism, Alkaloid biosynthesis I, Arginine and proline metabolism, Carbon fixation, Cysteine metabolism, Glutamate metabolism, Novobiocin biosynthesis, Phenylalanine metabolism, Phenylalanine, tyrosine and tryptophan biosynthesis, Tyrosine metabolism, immunology
64	7.03E-05	SLC6A12	6539	12p13	
65	7.29E-05	SLC16A2	6567	Xq13.2	Perou's- Intrinsic- Breast-Cancer-Genes

66	7.35E-05	MARCKSL1	65108	1p35.1	
67	7.72E-05	FKBP10	60681	17q21.2	
68	7.86E-05	DHTKD1	55526	10p14	
69	7.88E-05	ACSF2	80221	17q21.33	
70	8.25E-05	CYP2C8	1558	10q23.33	Arachidonic acid metabolism, Linoleic acid metabolism, Metabolism of xenobiotics by cytochrome P450
71	8.35E-05	ANP32B	10541	9q22.32	TACI and BCMA stimulation of B cell immune responses.
72	8.53E-05	UBE2S	27338	19q13.43	
73	8.88E-05	RAP1GAP	5909	1p36.1-p35	
74	8.97E-05	PYGL	5836	14q21-q22	Insulin signaling pathway, Starch and sucrose metabolism
75	8.97E-05	UGP2	7360	2p14-p13	Galactose metabolism, Nucleotide sugars metabolism, Pentose and glucuronate interconversions, Starch and sucrose metabolism
76	9.02E-05	PCK2	5106	14q12	Adipocytokine signaling pathway, Citrate cycle (TCA cycle), Insulin signaling pathway, PPAR signaling pathway, Pyruvate metabolism
77	9.03E-05	VEGFA	7422	6p12	Actions of Nitric Oxide in the Heart, Hypoxia-Inducible Factor in the Cardiovascular System, VEGF, Hypoxia, and Angiogenesis, Cytokine-cytokine receptor interaction, Focal adhesion, mTOR signaling pathway, VEGF signaling pathway
78	9.33E-05	SIAH2	6478	3q25	Perou's- Intrinsic- Breast-Cancer-Genes, Tryptophan metabolism
79	9.42E-05	HSD17B6	8630	12q13	
80	9.44E-05	CHST8	64377	19q13.1	Cysteine metabolism
81	9.50E-05	PCTP	58488	17q21-q24	
82	9.72E-05	ALAS1	211	3p21.1	Hemoglobin\'s Chaperone, Glycine, serine and threonine metabolism
83	9.77E-05	GRHPR	9380	9q12	Glyoxylate and dicarboxylate metabolism, Pyruvate metabolism
84	9.99E-05	LYST	1130	1q42.1-q42.2	
85	1.00E-04	RPS20	6224	8q12	Ribosome
86	0.0001	NR1I3	9970	1q23.3	Mechanism of Acetaminophen Activity and Toxicity, Nuclear Receptors in Lipid Metabolism and Toxicity
87	0.000101	PLEKHB1	58473	11q13.5-q14.1	
88	0.000102	HGD	3081	3q13.33	Styrene degradation, Tyrosine metabolism
89	0.000103	F12	2161	5q33-qter	Intrinsic Prothrombin Activation Pathway, Complement and coagulation cascades, immunology

90	0.000103	PKM2	5315	15q22	Carbon fixation, Glycolysis / Gluconeogenesis, Insulin signaling pathway, Purine metabolism, Pyruvate metabolism, Type II diabetes mellitus
91	0.000106	KLHL26	55295	19p13.11	
92	0.000106	C14orf105	55195	14q23.1	
93	0.000106	SMARCA2	6595	9p22.3	Folate biosynthesis, Starch and sucrose metabolism, development, gene_regulation, transcription
94	0.000106	HIST1H4C	8364	6p21.3	
95	0.000106	FLRT3	23767	20p11	
96	0.000108	APOL6	80830	22q12.3	
97	0.000112	LPCAT1	79888	5p15.33	
98	0.000114	CXCL10	3627	4q21	Cytokine-cytokine receptor interaction, Toll-like receptor signaling pathway
99	0.000116	KHDRBS1	10657	1p32	Regulation of Splicing through Sam68
100	0.000125	NDUFS7	374291	19p13.3	Oxidative phosphorylation
101	0.000127	C12orf48	55010	12q23.2	
102	0.000129	AGXT2L1	64850	4q25	
103	0.00013	SC65	10609	17q21.2	
104	0.000131	SECTM1	6398	17q25	
105	0.000133	METTL7A	25840	12q13.13	
106	0.000137	PTPRT	11122	20q12-q13	
107	0.000138	F13B	2165	1q31-q32.1	Complement and coagulation cascades, immunology
108	0.000139	NR1I2	8856	3q12-q13.3	Nuclear Receptors in Lipid Metabolism and Toxicity
109	0.000143	FETUB	26998	3q27	
110	0.000145	NT5DC2	64943	3p21.1	
111	0.00015	TOB1			Role of Tob in T-cell activation
112	0.000154	FNDC3B	64778	3q26.31	
113	0.000155	SULT4A1	25830	22q13.2-q13.31	Cysteine metabolism
114	0.000155	PLCG2	5336	16q24.1	B cell receptor signaling pathway, Calcium signaling pathway, Epithelial cell signaling in Helicobacter pylori infection, Fc epsilon RI signaling pathway, Inositol phosphate metabolism, Leukocyte transendothelial migration, Natural killer cell mediated cytotoxicity, Phosphatidylinositol signaling system, VEGF signaling pathway, cell_signaling, signal_transduction
115	0.000157	METTL9	51108	16p13-p12	
116	0.000159	SMTN	6525	22q12.2	
117	0.000163	PPFIA4	8497	1q32.1	

118	0.000166	EI24	9538	11q24	
119	0.000168	CYP4A11			Nuclear Receptors in Lipid Metabolism and Toxicity, Arachidonic acid metabolism, Fatty acid metabolism, PPAR signaling pathway, pharmacology
120	0.000169	MARCKS			Effects of calcineurin in Keratinocyte Differentiation
121	0.000175	ADM	133	11p15.4	angiogenesis
122	0.000176	ATP6V0E2	155066	7q36.1	
123	0.000178	PQLC1	80148	18q23	
124	0.000178	CENPM	79019	22q13.2	
125	0.000179	TMEM140	55281	7q33	
126	0.000181	CARD10	29775	22q13.1	
127	0.000183	ZNF84	7637	12q24.33	transcription
128	0.000183	CA9	768	9p13-p12	Nitrogen metabolism
129	0.000184	CPN2	1370	3q29	
130	0.000186	MPDZ	8777	9p24-p22	Tight junction
131	0.000189	GLG1	2734	16q22-q23	Cell adhesion molecules (CAMs)
132	0.000195	COX17	10063	3q13.33	Oxidative phosphorylation, misc
133	0.000197	NDRG1			
134	0.000198	KIAA0564	23078	13q14.11	
135	0.000199	GSTK1	373156		Glutathione metabolism, Metabolism of xenobiotics by cytochrome P450
136	0.000201	CCL25	6370	19p13.2	Cytokine-cytokine receptor interaction
137	0.000202	REPS2	9185	Xp22.2	Perou's- Intrinsic- Breast-Cancer-Genes
138	0.000206	MCM5	4174	22q13.1	CDK Regulation of DNA Replication, Cell cycle
139	0.000207	MFAP2	4237	1p36.1-p35	immunology
140	0.000211	ABCA6	23460	17q24.3	
141	0.000211	EPHX2	2053	8p21-p12	Arachidonic acid metabolism, Tetrachloroethene degradation, immunology, metabolism, pharmacology
142	0.000215	C16orf45	89927	16p13.11	Perou's- Intrinsic- Breast-Cancer-Genes
143	0.000225	EPHX1	2052	1q42.1	Eicosanoid Metabolism, Metabolism of xenobiotics by cytochrome P450, immunology, metabolism, pharmacology
144	0.000228	GNMT	27232	6p12	Glycine, serine and threonine metabolism
145	0.00023	ACADM	34	1p31	beta-Alanine metabolism, Fatty acid metabolism, PPAR signaling pathway, Propanoate metabolism, Valine, leucine and isoleucine degradation, immunology
146	0.000233	DUSP6			Regulation of MAP Kinase Pathways Through Dual Specificity Phosphatases, MAPK signaling pathway
147	0.000239	C7orf10	79783	7p14.1	

148	0.000243	CLEC1A	51267	12p13.2	
149	0.000247	LAMB1	3912	7q22	Prion Pathway, Cell Communication, ECM-receptor interaction, Focal adhesion, Prion disease
150	0.000248	HNRPH3			
151	0.000251	CUX2	23316	12q24.11-q24.12	
152	0.000253	KRT4	3851	12q12-q13	Cell Communication, immunology
153	0.000254	DDAH2	23564	6p21.3	
154	0.000254	NDFIP1	80762	5q31.3	
155	0.000257	GLYAT	10249	11q12.1	Malate-aspartate shuttle
156	0.00026	FGGY	55277	1p32.1	
157	0.000267	MFSD10	10227	4p16.3	
158	0.000267	PLAG1	5324	8q12	
159	0.000275	PEBP1	5037	12q24.23	Signal transduction through IL1R
160	0.000275	NECAB2	54550	16q23.3	
161	0.000288	MTHFD1	4522	14q24	Glyoxylate and dicarboxylate metabolism, One carbon pool by folate
162	0.000291	LOC120364			
163	0.000291	MORC3	23515	21q22.13	
164	0.000293	BHMT2	23743	5q13	
165	0.000294	DAAM2	23500	6p21.2	Wnt signaling pathway
166	0.000295	CREBL2	1389	12p13	
167	0.000296	MCCC2	64087	5q12-q13	Valine, leucine and isoleucine degradation
168	0.000297	LIMS1			
169	0.000298	XRCC6	2547	22q13.2-q13.31	Telomeres, Telomerase, Cellular Aging, and Immortality
170	0.000299	SERPINH1	871	11q13.5	
171	0.000302	PROSC	11212	8p11.2	
172	0.000305	RPL9			Ribosome
173	0.00031	SORD			Fructose and mannose metabolism, metabolism
174	0.000314	PHLDA2	7262	11p15.5	
175	0.00032	KRT8P12	90133	3q26.1	
176	0.00032	PCCB	5096	3q21-q22	Propanoate metabolism, Valine, leucine and isoleucine degradation, immunology, metabolism

177	0.00032	MAPK13	5603	6p21.31	Keratinocyte Differentiation, MAPKinase Signaling Pathway, Stathmin and breast cancer resistance to antimicrotubule agents, Epithelial cell signaling in Helicobacter pylori infection, Fc epsilon RI signaling pathway, GnRH signaling pathway, Leukocyte transendothelial migration, MAPK signaling pathway, Toll-like receptor signaling pathway, VEGF signaling pathway
178	0.000322	ADCY9	115	16p13.3	Calcium signaling pathway, Gap junction, GnRH signaling pathway, Purine metabolism
179	0.000326	FAIM	55179	3q22.3	
180	0.000329	PLCB1	23236	20p12	Activation of PKC through G protein coupled receptor, Aspirin Blocks Signaling Pathway Involved in Platelet Activation, Cadmium induces DNA synthesis and proliferation in macrophages, CCR3 signaling in Eosinophils, Eicosanoid Metabolism, fMLP induced chemokine gene expression in HMC-1 cells, G-Protein Signaling Through Tubby Proteins, Phospholipase C Signaling Pathway, Phospholipids as signalling intermediaries, PKC-catalyzed phosphorylation of inhibitory phosphoprotein of myosin phosphatase, Regulation of ck1/cdk5 by type 1 glutamate receptors, Role of ß-arrestins in the activation and targeting of MAP kinases, Roles of ß-arrestin-dependent Recruitment of Src Kinases in GPCR Signaling, ß-arrestins in GPCR Desensitization, Thrombin signaling and protease-activated receptors, Calcium signaling pathway, Gap junction, GnRH signaling pathway, Inositol phosphate metabolism, Long-term depression, Long-term potentiation, Phosphatidylinositol signaling system, Wnt signaling pathway
181	0.00033	SPP1	6696	4q21-q25	Regulators of Bone Mineralization, Cell Communication, ECM-receptor interaction, Focal adhesion, immunology
182	0.00033	SAR1B	51128	5q31.1	
183	0.000331	CRYL1	51084	13q12.11	
184	0.000335	PINK1	65018	1p36	Neurodegenerative Disorders, Parkinson\'s disease
185	0.000336	TTR	7276	18q12.1	immunology
186	0.000343	N4BP2L1	90634	13q12-q13	
187	0.000345	CYB5A	1528	18q23	Perou's- Intrinsic- Breast-Cancer-Genes
188	0.000345	DNASE1L3	1776	3p21.1-p14.3	gene_regulation, immunology

189	0.000352	IRF2	3660	4q34.1-q35.1	gene_regulation, transcription
190	0.00036	ACSM1	116285	16p12.2	Butanoate metabolism
191	0.000364	CDC37L1	55664	9p24.1	
192	0.000367	FAM60A	58516	12p11	
193	0.000368	LOC388152	388152	15q25.2	
194	0.000376	ALDH7A1	501	5q31	Arginine and proline metabolism, Ascorbate and aldarate metabolism, beta-Alanine metabolism, Bile acid biosynthesis, Butanoate metabolism, Fatty acid metabolism, Glycerolipid metabolism, Glycolysis / Gluconeogenesis, Histidine metabolism, Limonene and pinene degradation, Lysine degradation, Propanoate metabolism, Pyruvate metabolism, Tryptophan metabolism, Valine, leucine and isoleucine degradation
195	0.000379	RPS3	6188	11q13.3-q13.5	Ribosome
196	0.000379	PSME2			Antigen processing and presentation
197	0.000385	DGCR9	25787	22q11.21	
198	0.000386	MSRA	4482	8p23.1	
199	0.000387	SERPINC1	462	1q23-q25.1	Acute Myocardial Infarction, Extrinsic Prothrombin Activation Pathway, Intrinsic Prothrombin Activation Pathway, Complement and coagulation cascades
200	0.000391	PTPN18	26469	2q21.1	
201	0.000393	HIGD2A	192286	5q35.2	
202	0.000399	PTP4A3	11156	8q24.3	
203	0.000401	FAHD2A	51011	2p24.3-p11.2	
204	0.000405	FBLN1	2192	22q13.31	immunology
205	0.000405	CBR1	873	21q22.13	Perou's- Intrinsic- Breast-Cancer-Genes, Arachidonic acid metabolism, immunology
206	0.000406	RCL1	10171	9p24.1-p23	
207	0.000409	ECHS1	1892	10q26.2-q26.3	Benzoate degradation via CoA ligation, beta-Alanine metabolism, Butanoate metabolism, Caprolactam degradation, Fatty acid elongation in mitochondria, Fatty acid metabolism, Limonene and pinene degradation, Lysine degradation, Propanoate metabolism, Tryptophan metabolism, Valine, leucine and isoleucine degradation
208	0.000419	KPNA1	3836	3q21	immunology
209	0.000425	RNF24	11237	20p13-p12.1	
210	0.000427	C1S	716	12p13	Classical Complement Pathway, Complement Pathway, Complement and coagulation cascades, immunology

211	0.000429	C6orf97	80129	6q25.1	
212	0.000436	KIR3DX1	90011	19q13.42	
213	0.000443	LY86	9450	6p25.1	
214	0.000445	CRKL	1399	22q11|22q11.21	IL-2 Receptor Beta Chain in T cell Activation, Inhibition of Cellular Proliferation by Gleevec, Integrin Signaling Pathway, Links between Pyk2 and Map Kinases, Signaling of Hepatocyte Growth Factor Receptor, Focal adhesion, Insulin signaling pathway, MAPK signaling pathway, Regulation of actin cytoskeleton, tsonc
215	0.000449	KCNH7	90134	2q24.2	
216	0.000457	STARD5	80765	15q26	
217	0.000458	CD4	920	12pter-p12	Activation of Csk by cAMP-dependent Protein Kinase Inhibits Signaling through the T Cell Receptor, Antigen Dependent B Cell Activation, Bystander B Cell Activation, Cytokines and Inflammatory Response, HIV Induced T Cell Apoptosis, HIV-1 defeats host-mediated resistance by CEM15, IL 17 Signaling Pathway, IL 5 Signaling Pathway, Lck and Fyn tyrosine kinases in initiation of TCR Activation, NO2-dependent IL 12 Pathway in NK cells, Regulation of hematopoiesis by cytokines, Selective expression of chemokine receptors during T-cell polarization, T Helper Cell Surface Molecules, Antigen processing and presentation, Cell adhesion molecules (CAMs), Hematopoietic cell lineage, T cell receptor signaling pathway, angiogenesis, immunology, metastasis
218	0.000459	VGLL4	9686	3p25.2	
219	0.00046	TRIO	7204	5p15.1-p14	Rac 1 cell motility signaling pathway
220	0.00046	KIAA0500	57237	14q32.2	
221	0.000461	MAFF	23764	22q13.1	Oxidative Stress Induced Gene Expression Via Nrf2
222	0.000462	HK2			Aminosugars metabolism, Fructose and mannose metabolism, Galactose metabolism, Glycolysis / Gluconeogenesis, Starch and sucrose metabolism, Streptomycin biosynthesis, immunology, misc
223	0.000468	HSD17B8	7923	6p21.3	Androgen and estrogen metabolism
224	0.000469	CES1	1066	16q13-q22.1	Alkaloid biosynthesis II, immunology
225	0.000473	WHSC1	7468	4p16.3	
226	0.000476	SRD5A1	6715	5p15	Androgen and estrogen metabolism, Bile acid biosynthesis

227	0.000479	FUCA1	2517	1p34	Glycan structures - degradation, N-Glycan degradation, immunology
228	0.000484	GTSE1	51512	22q13.2-q13.3	
229	0.000484	C8orf70			
230	0.000489	CTSO	1519	4q31-q32	
231	0.00049	PLEKHA1	59338	10q26.13	
232	0.00049	DBN1	1627	5q35.3	
233	0.000496	HAO1	54363	20p12	Glyoxylate and dicarboxylate metabolism
234	0.000497	FAM64A	54478	17p13.2	
235	0.000499	PHF21A	51317	11p11.2	
236	0.000506	ALDH6A1	4329	14q24.3	Inositol metabolism, Propanoate metabolism, Valine, leucine and isoleucine degradation
237	0.000511	TRSPAP1	54952	1p35.3	
238	0.000519	STC1	6781	8p21-p11.2	
239	0.000521	HMGA2	8091	12q15	
240	0.000523	KLRB1	3820	12p13	
241	0.000529	ALDH5A1	7915	6p22.2-p22.3	Arginine and proline metabolism, Butanoate metabolism, Glutamate metabolism
242	0.000532	COX7B	1349	Xq21.1	Oxidative phosphorylation
243	0.000539	GOLM1	51280	9q21.33	
244	0.000542	CKAP4	10970	12q23.3	
245	0.000544	CYSLTR2			Eicosanoid Metabolism, Calcium signaling pathway, Neuroactive ligand-receptor interaction
246	0.000544	SSRP1	6749	11q12	
247	0.000547	HMG2L1	10042	22q13.1	
248	0.00055	NES	10763	1q23.1	Cell Communication
249	0.000551	CSPP1	79848	8q13.2	
250	0.000558	UCK2	7371	1q23	Pyrimidine metabolism
251	0.000559	SLC25A20	788	3p21.31	
252	0.000559	RPL37A	6168	2q35	Ribosome
253	0.000568	GAL3ST1	9514	22q12.2	Sphingolipid metabolism
254	0.000569	BDH1	622	3q29	Butanoate metabolism, Synthesis and degradation of ketone bodies
255	0.000579	DEPDC5	9681	22q12.3	
256	0.00059	MYCN	4613	2p24.1	tsonc
257	0.000592	CNTLN	54875	9p22.2	
258	0.000599	SLC22A7	10864	6p21.2-p21.1	
259	0.000599	CYP2A6	1548	19q13.2	Perou's- Intrinsic- Breast-Cancer-Genes, immunology, pharmacology

260	0.000605	UGT2B15	7366	4q13	Androgen and estrogen metabolism, Metabolism of xenobiotics by cytochrome P450, Pentose and glucuronate interconversions, Porphyrin and chlorophyll metabolism, Starch and sucrose metabolism, pharmacology
261	0.000605	NDUFA3	4696	19q13.42	Oxidative phosphorylation
262	0.000607	CLMN	79789	14q32.13	
263	0.000608	SEC31B	25956	10q24.31	
264	0.000613	C11orf21	29125	11p15.5	
265	0.000615	TOM1L1	10040	17q23.2	
266	0.000617	BTD	686	3p25	Biotin metabolism, immunology
267	0.00062	RDH16	8608	12q13.3	
268	0.000624	MAOA	4128	Xp11.3	Arginine and proline metabolism, Glycine, serine and threonine metabolism, Histidine metabolism, Phenylalanine metabolism, Tryptophan metabolism, Tyrosine metabolism, behavior, misc
269	0.000628	DAO	1610	12q24	Arginine and proline metabolism, D-Arginine and D-ornithine metabolism, Glycine, serine and threonine metabolism, immunology
270	0.000629	KIF11	3832	10q24.1	
271	0.000634	TRIM21	6737	11p15.5	
272	0.000635	IL2RB	3560	22q13|22q13.1	IL 2 signaling pathway, IL-2 Receptor Beta Chain in T cell Activation, Cytokine-cytokine receptor interaction, Jak-STAT signaling pathway, immunology
273	0.000638	PDE1B	5153	12q13	Calcium signaling pathway, Purine metabolism
274	0.000643	EPO	2056	7q22	EPO Signaling Pathway, Erythrocyte Differentiation Pathway, Erythropoietin mediated neuroprotection through NF-kB, Hypoxia-Inducible Factor in the Cardiovascular System, Regulation of hematopoiesis by cytokines, Cytokine-cytokine receptor interaction, Hematopoietic cell lineage, Jak-STAT signaling pathway, immunology
275	0.000643	SAMHD1	25939	20pter-q12	
276	0.000645	ACBD4	79777	17q21.31	
277	0.00065	OASL	8638	12q24.2	
278	0.000663	GNL3	26354	3p21.1	
279	0.000664	CLDN15	24146	7q11.22	Cell adhesion molecules (CAMs), Leukocyte transendothelial migration, Tight junction
280	0.000668	SLC38A1	81539	12q13.11	
281	0.000675	DACH1	1602	13q22	

282	0.000677	DHRS4			Arachidonic acid metabolism
283	0.000686	Sep 09			
284	0.000691	MCAM	4162	11q23.3	
285	0.000699	NPM3	10360	10q24.31	
286	0.000702	TRIB3	57761	20p13-p12.2	
287	0.000705	PUS1	80324	12q24.33	
288	0.000706	FLJ20712	55025	7p14.3	
289	0.000711	SDHD	6392	11q23	Electron Transport Reaction in Mitochondria, Citrate cycle (TCA cycle), Oxidative phosphorylation
290	0.000718	SLC2A2	6514	3q26.1-q26.2	Maturity onset diabetes of the young, Type II diabetes mellitus, immunology
291	0.00072	ERCC8	1161	5q12.1	
292	0.00072	SLC6A1	6529	3p25-p24	
293	0.000722	PAFAH1B3	5050	19q13.1	Glycerophospholipid metabolism
294	0.000729	BZW2	28969	7p21.1	
295	0.000733	TRIM22			
296	0.00074	TUBB			Gap junction, immunology
297	0.00075	ACAA2			Benzoate degradation via hydroxylation, Bile acid biosynthesis, Fatty acid elongation in mitochondria, Fatty acid metabolism, Valine, leucine and isoleucine degradation
298	0.000751	STRN	6801	2p22-p21	
299	0.000751	IRF9	10379	14q11.2	Bone Remodelling, IFN alpha signaling pathway, Jak-STAT signaling pathway
300	0.000756	PTDSS2	81490	11p15	Glycerophospholipid metabolism
301	0.000769	C9orf46	55848	9p24.1	
302	0.00077	RPL7	6129	8q21.11	Ribosome
303	0.000776	PES1	23481	22q12.1	
304	0.000802	APBA1	320	9q13-q21.1	Neurodegenerative Disorders
305	0.000811	CYP4F3	4051	19p13.2	Arachidonic acid metabolism, pharmacology
306	0.000822	ACOX2	8309	3p14.3	Perou's- Intrinsic- Breast-Cancer-Genes, Fatty acid metabolism, PPAR signaling pathway
307	0.000823	HAAO	23498	2p21	Tryptophan metabolism
308	0.00083	MKI67	4288	10q25-qter	cell_cycle, gene_regulation, transcription
309	0.000831	ADH6	130	4q23	1- and 2-Methylnaphthalene degradation, Bile acid biosynthesis, Fatty acid metabolism, Glycerolipid metabolism, Glycolysis / Gluconeogenesis, Metabolism of xenobiotics by cytochrome P450, Tyrosine metabolism
310	0.00084	C14orf108	55745	14q23.1	
311	0.000841	CES2	8824	16q22.1	

312	0.000842	GADD45A	1647	1p31.2-p31.1	ATM Signaling Pathway, Cell Cycle: G2/M Checkpoint, Hypoxia and p53 in the Cardiovascular system, p53 Signaling Pathway, Cell cycle, MAPK signaling pathway
313	0.000842	MFSD1	64747	3q25.33	
314	0.000848	C14orf115	55237	14q24.3	
315	0.000856	MLYCD	23417	16q24	beta-Alanine metabolism, Propanoate metabolism
316	0.000865	SLCO2B1	11309	11q13	
317	0.000867	ACAD8	27034	11q25	1- and 2-Methylnaphthalene degradation, Bile acid biosynthesis
318	0.00087	HLF	3131	17q22	gene_regulation, transcription
319	0.000879	MYO1B	4430	2q12-q34	
320	0.000902	MUT	4594	6p12.3	Catabolic Pathways for Methionine, Isoleucine, Threonine and Valine, Propanoate metabolism, Valine, leucine and isoleucine degradation, immunology
321	0.000904	STAG3	10734	7q22.1	
322	0.000909	SLC22A1	6580	6q26	
323	0.000913	RGN	9104	Xp11.3	
324	0.000913	LAMA4	3910	6q21	Cell Communication, ECM-receptor interaction, Focal adhesion, angiogenesis
325	0.000916	WSB1	26118	17q11.1	
326	0.000919	FBXO7	25793	22q12-q13	
327	0.000928	GPR107	57720	9q34.11	
328	0.000948	G6PD			Glutathione metabolism, Pentose phosphate pathway, immunology
329	0.000952	PFKFB1	5207	Xp11.21	Fructose and mannose metabolism
330	0.000953	ANGPT2	285	8p23.1	angiogenesis
331	0.000955	LOC441454			
332	0.000962	CBWD2			
333	0.000968	PPFIBP1	8496	12p11.23-p11.22	
334	0.000994	KLKB1	3818	4q34-q35	Intrinsic Prothrombin Activation Pathway, Complement and coagulation cascades, immunology
335	0.000994	NOX5	79400	15q23	
336	0.000995	CYP3A4	1576	7q21.1	Mechanism of Acetaminophen Activity and Toxicity, Nuclear Receptors in Lipid Metabolism and Toxicity, gamma-Hexachlorocyclohexane degradation, Linoleic acid metabolism, Metabolism of xenobiotics by cytochrome P450

References:

[1] Q.H. Ye, L.X. Qin, M. Forgues, P. He, J.W. Kim, A.C. Peng, R. Simon, Y. Li, A.I. Robles, Y. Chen, Z.C. Ma, Z.Q. Wu, S.L. Ye, Y.K. Liu, Z.Y. Tang, and X.W. Wang, Predicting hepatitis B virus-positive metastatic hepatocellular carcinomas using gene expression profiling and supervised machine learning. Nature medicine 9 (2003) 416-23.
